# The relationship between impulsivity and shame and guilt proneness on the prediction of internalizing and externalizing behaviors

**DOI:** 10.1016/j.heliyon.2019.e02746

**Published:** 2019-11-14

**Authors:** Helen Sanchez, D. Angus Clark, Sherecce A. Fields

**Affiliations:** Texas A&M University, USA

**Keywords:** Psychology, Clinical psychology, Impulsivity, Shame, Guilt, Mediation, Health-risk behavior

## Abstract

Shame and guilt are responses to moral transgressions that are characterized by negative self-evaluations and negative behavioral-evaluations, respectively. Previous research has found shame to be the more maladaptive of these “self-conscious” emotions due to its association with various health-risk behaviors. In the current study, undergraduate participants (n = 199) from a large, public university completed behavioral and self-report measures of impulsivity, shame and guilt-proneness, and behavioral tendencies. Exploratory factor analysis and mediation models were used to determine if shame and/or guilt-proneness significantly mediate the relationship between impulsivity and internalized/externalized problems. Findings demonstrate that impulsivity and shame proneness both positively predict internalized and externalized problem behavior, but indirect effects of shame and guilt are not significant. These findings indicate that shame and guilt do not reliably mediate the relationship between impulsivity and problem behavior, but they do support previous findings on the maladaptive nature of impulsivity and shame. Implications for the protective nature of guilt proneness are also discussed.

## 1 Introduction

### 1.1 Impulsivity

Impulsivity can be defined as a predisposition towards unplanned or rapid reactions to stimuli without the consideration of possible negative consequences (De Wit, 2009). Impulsivity is a multidimensional construct, encompassing traits such as impulsive decision-making, inattention, and disinhibition (Fields et al., 2009). Extensive research has demonstrated the associations between impulsivity and externalizing health risk behaviors such as substance use (Jentsch et al., 2014), binge eating (Dawe and Loxton., 2004), sexual risk-taking (Hoyle et al., 2000) and self-injury (Hamza et al., 2015). The relationship between impulsivity and internalized behavior is less straightforward. Previous studies have shown that low-impulsivity children are more prone to internalization and sadness than their high-impulsivity peers (Eisenberg et al., 2001; Eisenberg et al., 2009). However, studies of adults demonstrate links between depression and high impulsivity (Corruble et al., 1999; Grano et al., 2007). Additionally, high attentional, motor, and non-planning impulsivity on the Barratt Impulsiveness Scale (BIS-11; Patton et al., 1995) has been noted in depressed subjects (Corruble et al., 2003).

### 1.2 Shame and guilt-proneness

Although sometimes used interchangeably, shame is defined as a global, negative feeling about oneself, while guilt is seen as a negative feeling about a specific behavior (Lewis, 1971). The negative-self-evaluation that underlies shame causes one to attribute transgressions to personal character flaws that are fixed or difficult to alter. In contrast, guilt stems from the belief that transgressions are the result of behavioral errors, which may be corrected. As a result, shame-proneness is often accompanied by maladaptive avoidance or withdrawal behaviors (e.g. leaving a situation), while guilt-proneness is accompanied by adaptive approach behaviors (e.g. initiating reparative action; Tangney, 1994; Wolf et al., 2010). This conceptualization of shame and guilt differs from others, such as the internal and external components of shame proposed by Gilbert (1997) and the different trait and state components of shame assessed in Turner's Experiential Shame Scale (Turner, 1998). For the purposes of this study, more narrow definitions are used to draw a clear distinction between self-focused negative affect and that which is behavior focused (Sanftner et al., 1995).

Various externalized behaviors such as substance use (Rahim and Patton, 2015), binge eating (Sanftner et al., 1995), sexual risk-taking (Gilliland et al., 2011), and aggression (Tangney et al., 1992) show positive associations with shame-proneness but negative or negligible associations with guilt-proneness. Shame and guilt have been linked to internalizing problems like depression and anxiety in both children and adults (Ferguson et al., 2000; Mills et al., 2015; Eisenberg, 2000). A study of female sexual assault survivors found that “internalizing” participants with PTSD produced the highest scores on the Internalized Shame Scale (ISS: Cook, 1988) and the highest rates of major depression compared to “externalizers” with PTSD and those with simple PTSD. Results of the ISS, which examines internalized shame in the form of feelings of inferiority, indicate that “internalizers” may incorporate aspects of traumatic incidents into their identity, resulting in depressive symptoms (Miller and Resick, 2007).

In summary, impulsivity, shame, and guilt are associated with similar behavioral tendencies. More so than guilt, shame and impulsivity appear to be positively correlated with various externalizing health risk behaviors such as substance use, disordered eating, and self-injury. Both of these factors also show some relation to internalizing behaviors like depression and anxiety, but the results seem more mixed, with internalization appearing more characteristic of those with low impulsivity (Eisenberg et al., 2009).

### 1.3 Current study

It is evident that impulsivity and shame- and guilt-proneness have been linked to similar behavioral tendencies, but little research has directly examined how these constructs relate to each other. The current study aims to examine whether relationships between impulsivity and behavioral tendencies are mediated by shame or guilt. We hypothesize that individuals who score highly on measures of impulsivity will demonstrate greater shame-proneness than guilt-proneness. This hypothesis was made because impulsivity and shame-proneness both appear to be maladaptive and correlate with similar externalized health-risk behaviors. Guilt appears to be more adaptive, so there is less indication that it will be associated with negative behaviors (Eisenberg, 2000). We also hypothesize that the relationship between impulsivity and problem outcomes will be mediated by shame and/or guilt.

An exploration of these relationships, and any clinically relevant findings, is warranted. If shame and/or guilt reliably mediate the proposed relationships, these findings could inform future behavioral interventions to address impulsivity. Impulsive individuals may experience shame and/or guilt as the result of externalized problem behaviors (e.g. substance abuse, binge eating). On the other hand, impulsive individuals may be motivated to engage in hedonic health-risk behaviors to relieve negative feelings of shame and/or guilt. Clinicians may find it relevant to explore whether internalizing and externalizing problems are motivated by, or used to cope with, shame and/or guilt.

## 2 Methods

### 2.1 Participants

Undergraduate students (n = 199) from a large, public university completed the study for course credit. 210 students were initially recruited but 11 were excluded from analyses after failing to complete all measures. All participants were required to be over 18 years of age and fluent in English. The majority of participants were white (79.5%), non-Hispanic (71.4%), and female (75.2%). 9 participants (4.52%) were in the clinical range for internalizing problems, and 8 participants (4.02%) met this threshold for externalizing problems. Informed consent was obtained prior to beginning study measures. The University IRB approved all aspects of this study.

### 2.2 Measures

#### 2.2.1 Delay discounting

Delay discounting was assessed utilizing an adjusting-delay task developed by Bickel & Colleagues (see Epstein et al., 2014 for description). Participants are presented with items prompting a choice between receiving a certain amount of money ($1000) after a specified delay (i.e., 1 day, 1 week, 1 month, 3 months, 1 year, 5 years, or 25 years) or receiving a smaller amount of money immediately. The value of the immediate reward was adjusted across trials by using a decreasing-adjustment algorithm. This procedure determined the point at which participants were indifferent to the difference between the smaller, immediate reward and the larger, delayed reward. An area under the curve (AUC) procedure, as specified by Myerson et al. (2001) was used to characterize data from the discounting task. From the AUC, smaller values indicate greater monetary discounting by delay and greater impulsivity. Johnson and Bickel (2002) found no difference in discounting rates between real and hypothetical rewards, supporting the validity of using of hypothetical rewards in delay discounting procedures. The delay discounting procedure has also shown strong test-retest reliability (Baker et al., 2003).

#### 2.2.2 Short UPPS-P impulsive behavior scale (SUPPS–P: Lynam, 2013)

The short UPPS-P is a 20-item self-report measure of impulsivity. Participants rate their agreement with statements on a 4-point Likert-type scale. The UPPS-P assesses impulsivity on five subscales, which include negative urgency, lack of premeditation, lack of perseverance, sensation seeking, and positive urgency. SUPPS-P subscales are strongly correlated with UPPS-P subscales, demonstrate adequate reliability, and allow time savings during administration of about 66% (Cyders et al., 2014).

#### 2.2.3 Adult Self-Report (ASR: Achenback and Rescorla, 2003)

The Adult Self-Report form is used to assess adult functioning according to DSM-oriented scales. The ASR provides information on substance use, aggressive behavior, depressive problems, anxiety problems, and adaptive functioning. The ASR has been found to have high test-retest reliability and content validity (Achenback and Rescorla, 2003). Participants completed 126 items from section VIII of the ASEBA Adult Self-Report form to evaluate for internalizing and externalizing problems. Portions I – VII of the form were omitted. The ASR has been shown to have adequate test-retest reliability (all *r*s above .71), good internal consistency, moderate cross-informant correlations, and substantial long-term score stability (*r =*.43-.53 over 10 years). The ASR manual also presents extensive evidence for content, criterion-related, and construct validity (Achenback and Rescorla, 2003).

#### 2.2.4 The Test of Self-Conscious Affect (TOSCA-3S: Tangney and Dearing, 2002)

The Test of Self-Conscious Affect (TOSCA-3S) is an 11-item, scenario-based measure of shame and guilt-proneness. Different scenarios of moral transgressions or adverse events were presented along with three possible responses to each. Participants rated how likely they were to engage in each response on a 5-point Likert-type scale. The TOSCA-3S provides six sub scores, which include shame-proneness, guilt-proneness, externalization, detachment-unconcern, alpha-pride, and beta-pride. This measure allows participants to rate an experience as eliciting both shame and guilt, so shared variance is analyzed. Shame and guilt subscores were used as mediators in the model. The short form has been previously shown to be adequately reliable and valid and to correlate highly with the longer shame (*r* = .94) and guilt (*r* = .93) subscales (Moyer et al., 2017; Tangney and Dearing, 2002).

### 2.3 Analytic strategy

Cross-sectional mediation models were specified to examine the associations between impulsivity, shame and guilt proneness, and pathological symptoms (Cheong and MacKinnon, 2012). In order to better capture the construct of impulsivity and take advantage of the multiple indicators collected (i.e. SUPPS-P subscales, DDQ), impulsivity was incorporated into these models as a latent variable^1^. Accordingly, prior to the main analyses a measurement model was established for impulsivity. The 6 impulsivity scales were initially included in an exploratory factor analysis (EFA) that used an oblique, geomin rotation. Results from the EFA were then used to inform the specification and evaluation of the confirmatory factor model that would be used in subsequent analyses.

**Fig. 1:**
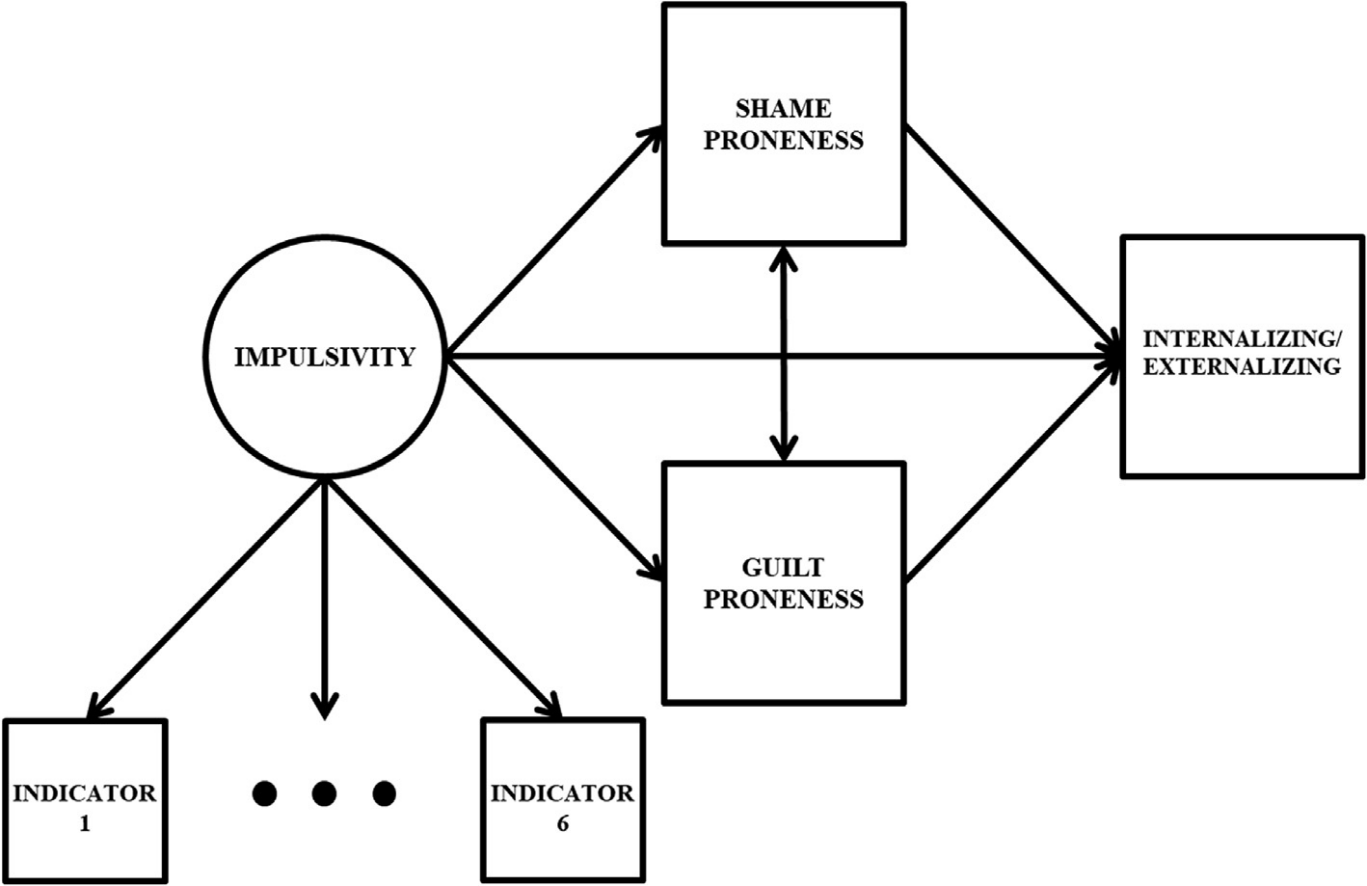
Latent Variable Double Mediation Model. Indicator 1 through Indicator 6 represent the six individual impulsivity measures that were used to specify the latent impulsivity factor. Mean structure and variances/residual variances omitted from figure for parsimony. A residual covariance -- not pictured -- was included between two factor indicator residual variances: lack of perseverance and lack of premeditation.

Once the optimal measurement model was identified, the primary mediation models of interest were specified. In these models both mediators (shame proneness and guilt proneness) and the outcome (either externalizing or internalizing problems) variables were regressed on the impulsivity factor. The outcome variable was also regressed on the mediator variables, and a covariance was specified between the two mediators. Both mediators were included simultaneously to provide a more holistic examination of the effects of interest, especially given past evidence regarding how shame and guilt often do not predict adjustment unless conditioning on the other (Paulhus et al., 2004). A simplified path diagram for this model is presented in Fig. 1. These models provided estimates of three major types of effects: the *direct effect* from the impulsivity factor to the outcome, the *indirect effects* from impulsivity to the outcome through the mediators (the product of the path from impulsivity to a specific mediator, and the path from that mediator to the outcome), and the *total effect*, which captures the holistic effect of all regression paths on the outcome (the sum of the total and indirect effects).

All analyses were conducted in Mplus version 8.2 (Muthen and Muthen, 2017) with full information maximum likelihood estimation. For the mediation models, 95% confidence intervals around the parameter estimates were calculated using Mplus’ percentile bootstrap procedure with 10,000 random draws (Falk, 2018). Separate models were run for each combination of mediator and outcome variable, for a total of 4 primary models.

## 3 Results

Descriptive statistics and the formulation of the measurement model are presented first. The primary models of interest are subsequently presented by outcome variable. A small set of exploratory follow-up analyses are also briefly discussed after the main analyses.

### 3.1 Preliminary analyses

Descriptive statistics for, and correlations between, all of the study variables are provided in Table 1. The intercorrelations between the impulsivity scales ranged from modest to strong in size (*r*s from .01 to .60; average *r* = .17). The EFA suggested that a 3 factor solution was optimal to account for the covariation between the impulsivity scales. This conclusion was informed by the converging evidence from a parallel analysis (with 1000 replications), an examination of the scree plot, and the change in model fit. Details of the 3 factor solution can be found in Table 2. Positive and negative urgency loaded strongly on one factor, lack of perseverance loaded on another factor, and sensation seeking and the delay discounting task (DDQ) loaded on the final factor. Lack of Premeditation failed to load robustly on any factor (i.e., all loadings less than .40).

**Table 1:** Correlations and descriptive statistics for study variables.

	1	2	3	4	5	6	7	8	9	10
1. Negative Urgency										
2. Positive Urgency	.60*									
3. Lack Perseverance	*.08*	*-.04*								
4. Lack Premeditation	*.10*	*.19*^†^	.50*							
5. Sensation Seeking	*.17*^†^	.32*	*-.14*^†^	*.01*						
6. DDQ	*-.03*	*-.11*	*-.03*	*-.01*	*-.10*					
7. Shame Proneness	.30*	.01	-.12	-.12	.05	.10				
8. Guilt Proneness	-.20^†^	-.31*	-.23^†^	-.25*	-.06	.13	.41*			
9. Internalizing	.42*	.20^†^	.18^†^	.06	.03	.03	.45*	-.06		
10. Externalizing	.40*	.29*	.11	.16^†^	.14	-.03	.28*	-.15^†^	.69*	
M	8.90	7.68	6.33	6.54	10.51	71.45	34.66	46.26	51.36	49.56
SD	3.02	2.60	1.89	1.99	2.75	24.95	8.72	7.23	12.93	11.39

Note. DDQ = delay discounting task; M = mean; SD = standard deviation. Intercorrelations between impulsivity measures italicized. * = *p* < .001; ^†^ = *p* < .05. The statistical significance level correcting for multiple comparisons -- 45 correlations in total -- was *p* < .001 (.05/45).

**Table 2:** Factor loadings from exploratory and confirmatory factor analyses.

	EFA		CFA	
	Factor 1	Factor 2	Un-STD	STD
Negative Urgency	.05	.55	.70	.60
Positive Urgency	.00	1.09	1.00	1.00
Lack Perseverance	2.06	.00	-.03	-.04
Lack Premeditation	.25	.18	.15	.19
Sensation Seeking	-.06	.29	.34	.32
DDQ	-.02	-.10	-1.01	-.11

Note. DDQ = delay discounting task; EFA = exploratory factor analysis; CFA = confirmatory factor analysis; Un-STD = unstandardized factor loadings; STD = standardized factor loadings. Oblique, geomin rotation used in EFA; factor correlation *r* = -.02.

Given that only one or two indicators characterized the factors in the EFA, a one-factor CFA was initially specified, with the factor mean and variance set to 0 and 1, respectively. This model provided a poor fit to the data by conventional standards (West et al., 2012): *χ*^*2*^ = 80.10, *df =* 10, *p* < .001; RMSEA = .19; SRMR = .11; CFI = .62; TLI = .43. In an attempt to balance acknowledgment of the EFA evidence for multidimensionality with the parsimony of a single factor model, a residual covariance was added between the lack of perseverance and lack of premeditation indicators. This specific residual covariance was chosen because of the relatively high observed correlation between these two indicators (*r =* .50), the corresponding conceptual overlap between these two scales (i.e., this residual covariance may simply reflect a method, or “nuisance” factor), and the fact that the lack of premeditation scale was the only other scale to load above .10 on the first factor (Table 2). This revised model was a good fit to the data by conventional standards: *χ*^*2*^ = 17.02, *df =* 9, *p* = .05; RMSEA = .07; SRMR = .05; CFI = .96; TLI = .93. Given the substantial difference in fit, this residual covariance was retained in the final measurement model. Importantly, the inclusion or omission of this residual covariance had little consequence for the parameters of note in the CFA; factor loadings and intercepts, and the factor mean and variance, were virtually unchanged across permutations.

Before specifying the mediation models, the identification of the factor was altered such that the factor mean and variance were allowed to freely estimate while the loading and intercept of the positive urgency scale were fixed to 1 and 0. Positive urgency was selected as the marker variable due to its exceptionally high loadings in the EFA and CFA models. This specification is equivalent to the previous model, but places the latent factor on the same metric as the marker variable (i.e., positive urgency). Thus, the impulsivity factor retains the scale of the SUPPS-P in the main analyses (the former specification was used to identify an optimal marker variable). The factor loadings (unstandardized and standardized) for this measurement model are presented in Table 2. Reflecting the results from the EFA, most loadings are not particularly strong. There is some variance common across all indicators, however it is only modestly related to most of the individual impulsivity scales.

### 3.2 Internalizing problems

**Fig. 2:**
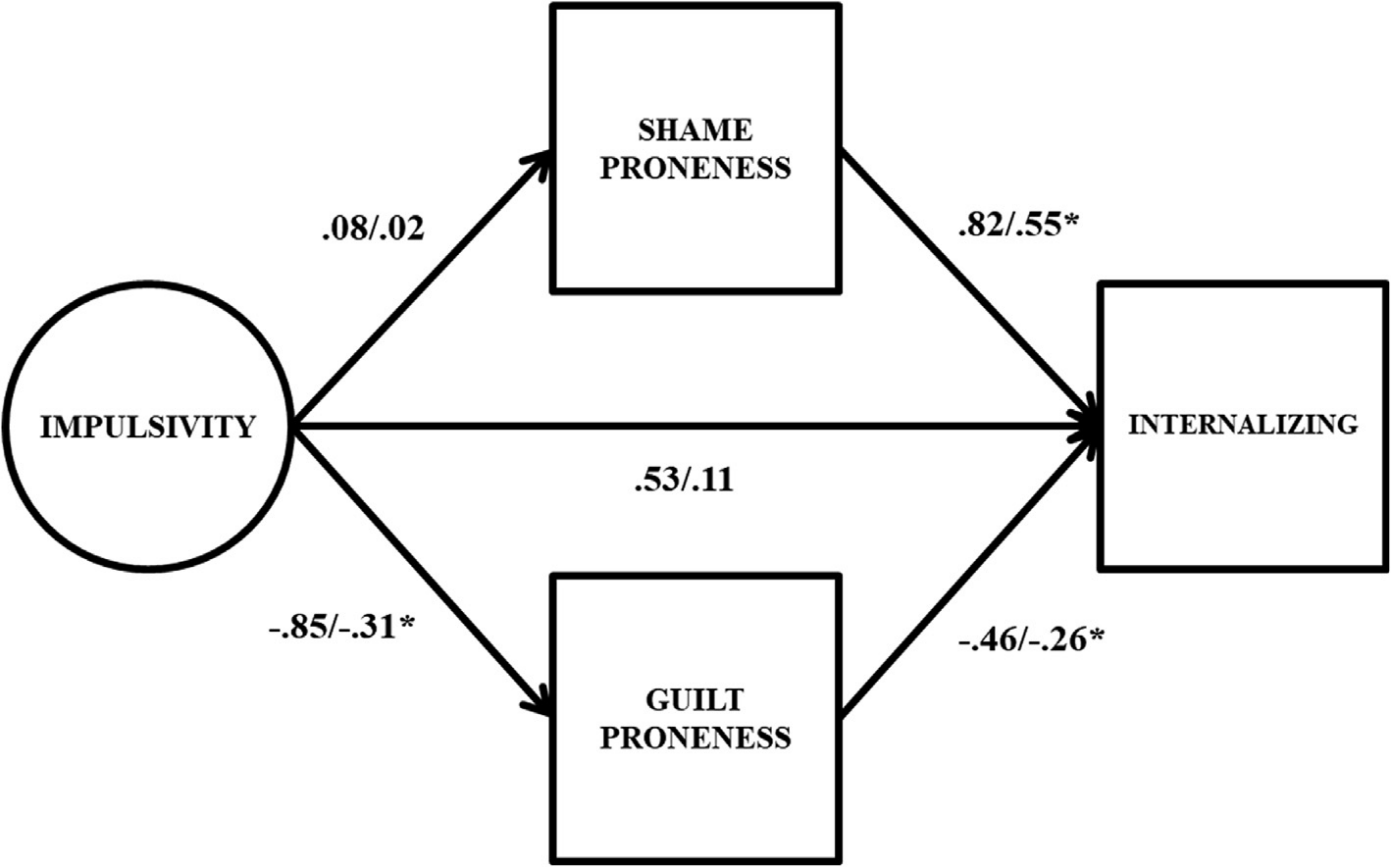
Path estimates from latent variable double mediation model predicting internalizing problems. Unstandardized coefficients to the left of the slash, standardized coefficients to the right of the slash; * = 95% confidence interval does not include 0. Factor indicators, mean structure, covariance between shame and guilt proneness, and variances/residual variances omitted from figure for parsimony.

Full results from the latent variable double mediation model are presented in Table 3. To facilitate the interpretation of this table, key path estimates are also presented graphically in Fig. 2. The direct effect from impulsivity to internalizing problems was statistically non-significant and small in size (β = .11). Impulsivity was a non-significant predictor of shame proneness (β = .02), but was moderately, significantly associated with guilt proneness (β = -.31). Finally, both shame (β = .55) and guilt (β = -.26) proneness were statistically significant predictors of internalizing problems.

**Table 3:** Results from latent variable double mediation models.

	Internalizing Problems			Externalizing Problems		
	Un-STD	95% CI	STD	Un-STD	95% CI	STD
Shame Proneness						
IMP → MED	.07	[-.39, .55]	.02	.09	[-.38, .56]	.03
MED → OUT	.82	[.62, 1.02]*	.55	.49	[.33, .66]*	.38
Indirect Effect	.06	[-.33, .46]	.01	.04	[-.18, .29]	.01
Guilt Proneness						
IMP → MED	-.85	[-1.31, -.42]*	-.31	-.85	[-1.31, -.42]*	-.31
MED → OUT	-.46	[-.72, -.15]*	-.26	-.37	[.33, .66]*	-.23
Indirect Effect	.39	[.09, .78]*	.08	.31	[.09, .60]*	.07
Direct and Total Effects						
Direct Effect	.53	[-.23, 1.27]	.11	.90	[.25, 1.56]*	.21
Total Indirect Effect	.45	[.05, .89]*	.09	.36	[.08, .66]*	.08
Total Effect	.98	[.21, 1.76]*	.20	1.26	[.54, 1.98]*	.29

Note. IMP → MED = path from impulsivity factor to mediator; MED → OUT = path from mediator to outcome; Un-STD = unstandardized coefficients; 95% CI = 95% confidence intervals for unstandardized coefficients; STD = standardized coefficients; * = *p* < .05. 95% confidence intervals derived via bias corrected bootstrap procedure with 10,000 random draws.

The indirect effect from impulsivity to internalizing problems through shame proneness was small and non-significant (β = .01). However, the indirect effect from impulsivity to internalizing problems through guilt proneness was statistically significant, though it was still small in magnitude (β = .08). This indirect effect implies that impulsivity in part predicts *higher* internalizing problems through its association with guilt proneness (while controlling for shame proneness); more impulsive individuals are less prone to guilt, and less guilt prone individuals report greater internalizing problems. The total indirect effect (i.e., the sum of the two individual indirect effects) was also statistically significant (β = .09), as was the total effect (β = .20).

### 3.3 Externalizing problems

**Fig. 3:**
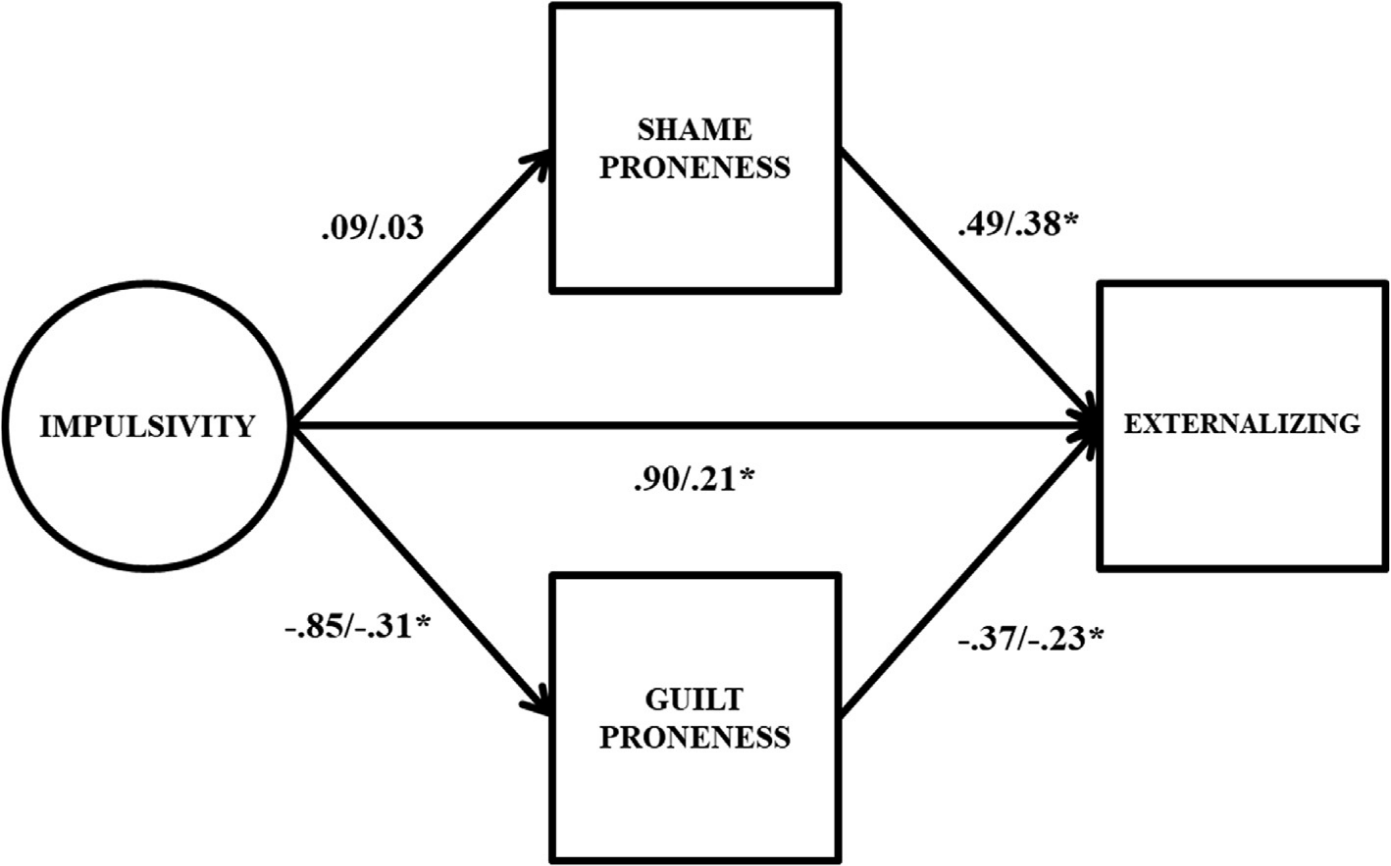
Path estimates from latent variable double mediation model predicting externalizing problems. Unstandardized coefficients to the left of the slash, standardized coefficients to the right of the slash; * = 95% confidence interval does not include 0. Factor indicators, mean structure, covariance between shame and guilt proneness, and variances/residual variances omitted from figure for parsimony.

Full results from the latent variable double mediation model are presented in Table 3. To facilitate the interpretation this table, key parameter estimates are also presented graphically in Fig. 3. The direct effect from impulsivity to externalizing problems was statistically significant and moderate in size (β = .21). Impulsivity was a non-significant predictor of shame proneness (β = .03), but was moderately, significantly associated with guilt proneness (β = -.31). Finally, both shame (β = .38) and guilt (β = -.23) proneness were statistically significant of externalizing problems.

The indirect effect from impulsivity to externalizing problems through shame proneness was small and not statistically significant (β = .01). However, the indirect effect from impulsivity to externalizing problems through guilt proneness was statistically significant, though it was still small in magnitude (β = .07). This indirect effect implies that impulsivity in part predicts *higher* externalizing problems through its association with guilt proneness (while controlling for shame proneness); more impulsive individuals are less prone to guilt, and less guilt prone individuals report greater externalizing problems. The total indirect effect was also statistically significant (β = .08), as was the total effect (β = .29).

### 3.4 Exploratory analyses

**Fig. 4:**
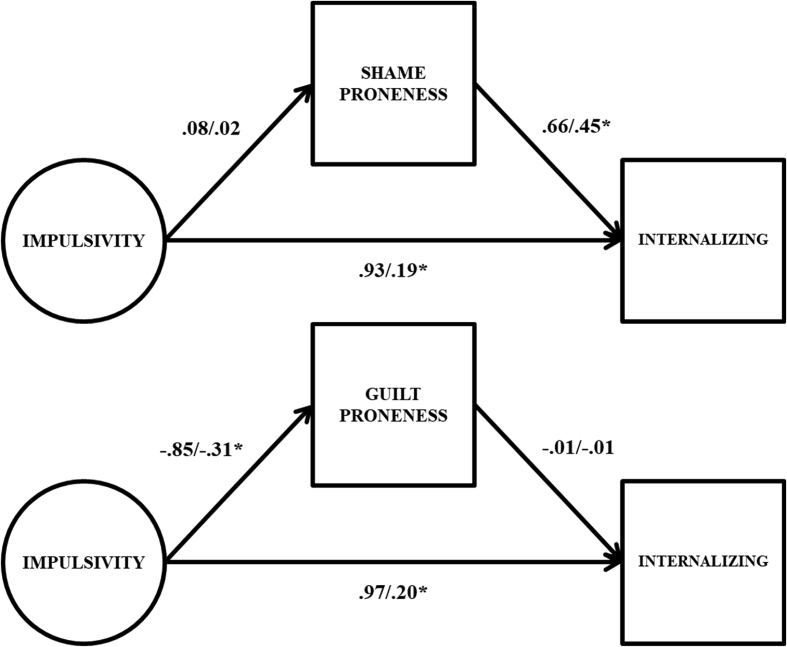
Path estimates from latent variable single mediation models predicting internalizing problems. Unstandardized coefficients to the left of the slash, standardized coefficients to the right of the slash; * = 95% confidence interval does not include 0. Factor indicators, mean structure, and variances/residual variances omitted from figure for parsimony.

**Fig. 5:**
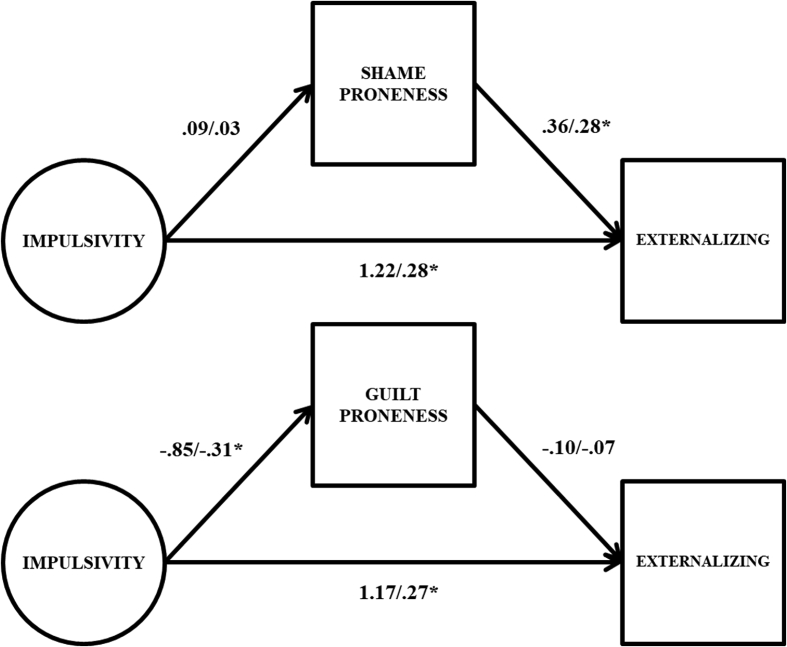
Path estimates from latent variable single mediation models predicting externalizing problems. Unstandardized coefficients to the left of the slash, standardized coefficients to the right of the slash; * = 95% confidence interval does not include 0. Factor indicators, mean structure, and variances/residual variances omitted from figure for parsimony.

Two different sets of exploratory follow up analyses were conducted. First, simpler single mediator latent mediation models were run in order to determine the importance of conditioning the outcomes on the other mediator given past findings regarding shame and guilt proneness (Paulhus et al., 2004). These results are presented in Table 4, and then graphically in Figs. 4 and 5. Results were mostly consistent with the main analyses, however, in contrast to the double mediator models the indirect effects running from impulsivity through guilt proneness to the outcomes were not statistically significant, and near 0 in size (and there was a statistically significant direct effect from impulsivity to internalizing problems). This suggests that the indirect effects observed in the main analyses are conditional on the inclusion of shame proneness in the model alongside guilt proneness. This is due to the fact that that guilt proneness was only associated with internalizing and externalizing problems after controlling for shame proneness. That is, among equally shame prone individuals more guilt proneness is associated with greater internalizing and externalizing problems, though guilt proneness by itself does not predict either problem dimension. This is emblematic of a suppressor situation, and is consistent with previous work on shame and guilt proneness showing that associations between shame and guilt, and adjustment, often do not emerge unless both predictors are included simultaneously (Paulhus et al., 2004) (see Table 5).

**Table 4:** Results from latent variable single mediation models.

	Internalizing Problems			Externalizing Problems		
	Un-STD	95% CI	STD	Un-STD	95% CI	STD
Shame Proneness						
IMP → MED	.08	[-.40, 55]	.02	.09	[-.38, .57]	.03
MED → OUT	.66*	[.47, .85]	.45	.36*	[.21, .52]	.28
Direct Effect	.93*	[.21, 1.68]	.19	1.22*	[.53, 1.80]	.28
Indirect Effect	.05	[-.26, .39]	.01	.03	[-.13, .23]	.01
Total Effect	.98*	[.22, 1.77]	.20	1.26*	[.54, 2.00]	.29
Guilt Proneness						
IMP → MED	-.85*	[-1.34, -.43]	-.31	-.85*	[-1.33, -.43]	-.31
MED → OUT	-.01	[-.29, .28]	-.01	-.10	[-.32, .13]	-.07
Direct Effect	.97*	[.16, 1.67]	.20	1.17*	[.45, 1.91]	.27
Indirect Effect	.01	[-.23, .25]	<.01	.09	[-.09, .30]	.02
Total Effect	.98*	[.22, 1.77]	.20	1.26*	[.54, 2.00]	.29

Note. IMP → MED = path from impulsivity factor to mediator; MED → OUT = path from mediator to outcome; Un-STD = unstandardized coefficients; 95% CI = 95% confidence intervals for unstandardized coefficients; STD = standardized coefficients; * = *p* < .05. 95% confidence intervals derived via bias corrected bootstrap procedure with 10,000 random draws.

**Table 5:** Standardized coefficients from single indicator double mediation models.

	Internalizing Problems						Externalizing Problems					
	NU	PU	LP	LPR	SS	DDQ	NU	PU	LP	LPR	SS	DDQ
Shame Proneness												
IMP → MED	.30*	.02	-.12	-.12	.05	.07	.30*	.02	-.11	-.12	.06	.08
MED → OUT	.46*	.55*	.57*	.57*	.57*	.57*	.29*	.38*	.41*	.42*	.40*	.41*
Indirect Effect	.14*	.01	-.07	-.07	.03	.04	.09*	.01	-.05	-.05	.02	.03
Guilt Proneness												
IMP → MED	-.20*	-.31*	-.23*	-.25*	-.06	.11	-.20*	-.31*	-.23*	-.25*	-.06	.12
MED → OUT	-.20*	-.26*	-.26*	-.45*	-.30*	-.30*	-.21*	-.23*	-.29*	-.28*	-.30*	-.31*
Indirect Effect	.04*	.08*	.06*	.07*	.02	-.03	.04*	.07*	.07*	.07*	.02	-.04
Direct and Total Effects												
Direct Effect	.25*	.11	.19*	.05	-.02	.04	.27*	.21*	.09	.14*	.10	.01
Total Indirect Effect	.18*	.09*	-.01	<.01	.05	.01	.13*	.08*	.02	.02	.04	<.01
Total Effect	.42*	.20*	.18*	.06	.03	.05	.40*	.29*	.11	.17*	.14*	.01

Note. NU = negative urgency; PU = positive urgency; LP = lack of perseverance; LPR = lack of premeditation; SS = sensation seeking; DDQ = delay discounting task; Single Mediator = latent variable mediation models in which only one mediators and indirect effects were specified; IMP → MED = path from impulsivity scale to mediator; MED → OUT = path from mediator to outcome; * = *p* < .05. Statistical significance based on 95% confidence intervals derived via bias corrected bootstrap procedure with 10,000 random draws.

Second, in light of the relatively modest degree of shared variance across the six impulsivity indicators a series of scale level double-mediator models were estimated. That is, mediation models were estimated in which the latent impulsivity factor was replaced by one of the six individual impulsivity scales. Each scale was considered in turn, and was included in two models, one for each outcome variable. Selected results are presented in Table 4. The findings across these models were largely consistent with what was reported in main analyses. That is, indirect effects were observed with guilt proneness in most models. The exception was that no indirect effects were observed with the sensation seeking scale or the DDQ. However, most associations between these variables and the others were particularly weak. Also, indirect effects were also observed with shame proneness and negative urgency for both outcomes. This should be interpreted with caution however given the number of models run and the fact that only two showed this effect.

## 4 Discussion

### 4.1 Current findings

Previous literature on shame and guilt-proneness has mainly focused on their correlation with health-risk behaviors without examining their relationships to impulsivity (VanDerhei et al., 2014; Levinson et al., 2016; Treeby and Bruno, 2012). The current study adds to the existing literature by exploring whether shame-proneness and/or guilt-proneness mediate the relationship between impulsivity and problematic behavioral outcomes. Our results illustrated a significant direct effect from impulsivity to externalizing problems but not internalizing problems. Shame demonstrated a significant direct effect with both internalizing and externalizing problems. This supports previous findings on the maladaptive nature of these constructs. Impulsivity is by definition a disregard for possible negative consequences, so its correlation with externalized problems is not surprising. Similarly, shame's correlation with health-risk behaviors, such as substance abuse (Dearing et al., 2005; Hequembourg and Dearing, 2013; Treeby and Bruno, 2012) and disordered eating (Sanftner et al., 1995; Levinson et al., 2016) has been well documented.

As expected, impulsivity was negatively related to guilt proneness in both double mediation models. This finding aligns with a previous study showing a lack of remorse or guilt among impulsive subjects following an impulsive decision task (Lin et al., 2009). Impulsive individuals may be less likely than those who are non-impulsive to negatively evaluate their behavior following a moral transgression, resulting in less guilt. On the other hand, individuals who are guilt-prone may inhibit risky impulses as a way to avoid resultant negative emotions. Additionally, indirect effects from impulsivity to internalizing and externalizing problems through its association with guilt were statistically significant. As mentioned previously, this indicates that impulsive individuals are less guilt-prone, and those who are less guilt-prone may demonstrate more internalizing and externalizing problems. This finding provides further support for the view of guilt as an adaptive response. As mentioned previously, guilt-proneness has been shown to correlate negatively or negligibly with problem behaviors such as substance use (Dearing et al., 2005), disordered eating (Sanftner et al., 1995), and self-injury (VanDerhei et al., 2014). However, these indirect effects were no longer significant in single mediator latent mediation models. Therefore, the seemingly adaptive qualities of guilt-proneness may only be apparent when controlling for shame-proneness.

While the processes underlying the link between impulsivity and self-conscious emotions are still somewhat unclear, the effect of negative emotion on impulsivity has been consistently noted. Whiteside and Lynam (2001) note that individuals high in urgency, a component of impulsivity, are more likely to act impulsively as a means to relieve negative emotions. Affective instability has also been proposed to underlie maladaptive behavior such as nonsuicidal self-injury, demonstrating the negative effects of “emotionally-driven” impulsivity (Peters et al., 2016). As negative affective experiences, shame and/or guilt may play a role in motivating impulsive behavior, which may provide a hedonic reward or relief from these negative emotions. On the other hand, the negative associations found between guilt and impulsivity may indicate that anticipation of a negative emotional state prevents some individuals from engaging in impulsive action. Similarly, previous work has shown a preventative effect of anticipated shame against impulsive consumer choices (Chun et al., 2007). Therefore, the link between impulsivity and shame and/or guilt may depend on whether the emotion is experienced prior to or following a behavior.

Internalizing and externalizing problems are categories that encompass a wide swath of maladaptive behaviors. The current findings highlight the importance of considering self-conscious emotions when designing behavioral interventions. Shame and guilt could be addressed within the context of treatments like Acceptance and Commitment Therapy (ACT). ACT has been used to treat internalizing issues such as depression and anxiety (Forman et al., 2007). It has also been used to address self-stigma, which is characteristic of externalizing issues such as polysubstance use (Luoma et al., 2008). While the current findings do not outline a novel treatment approach, they do support the use of treatments that address the role of shame, guilt, and other forms of negative self-evaluation in establishing maladaptive behavior patterns.

### 4.2 Limitations and further research

The current analysis is limited by the relative demographic homogeneity of the study sample (75.2% female, 79.5% white, 71.4% non-Hispanic). Due to an error on the demographics questionnaire, data about participant age is missing. All participants were required to be at least 18 years old and an undergraduate student to register for participation. As all participants were estimated to be age 18–25, the data reflects findings for an emerging-adult population. The uniform educational level and restricted age range of the sample further limit the generalizability of the results. The private, online format of the study resulted in several incomplete entries. Lack of interest or motivation to complete the survey may have led to random or inaccurate responses. Additionally, response bias may reduce the accuracy of the self-report measure, especially for items pertaining to substance abuse and other health risk behaviors. Lastly, although the size of the current sample should be adequate to detect mediated effects at least moderate in size (Fritz & MacKinnon, 2007), power is lower for detecting small effects. Accordingly, the general absence of mediation observed here could reflect a lack of power in addition to the true absence of indirect pathways. However, across many variables and analyses indirect effect sizes were consistently trivial, mostly below |.10|, suggesting that the contribution of any indirect pathways here are modest at best.

As mentioned previously, this study did not evaluate the internal and external subdivisions of shame proposed by Gilbert (1997). Internalized shame refers to inwardly focused attention and judgments about the self, while externalized shame describes the feeling of being perceived negatively by others (Gilbert and Andrews, 1998). According to this theorization, the self-critical thoughts evaluated by the TOSCA-3S may only describe internal shame. While the general shame score, as evaluated by the TOSCA-3S, did not significantly mediate the relationship between impulsivity and behavior problems, internal and external shame may have shown different relationships to impulsivity. The inclusion of additional measures of shame and guilt such as the Harder Personal Feelings Questionnaire (PFQ-2, Harder and Zalma, 1990) and the Internalized Shame Scale (Cook, 1988) could be used to examine how specific components of these emotions (e.g. internal and external shame) relate to impulsivity.

Further research should examine the relationship between impulsivity and self-conscious emotions in children and older adults. Emerging adults’ lack of both parental supervision and complete adult responsibilities may make them less likely to evaluate consequences of risky behavior than both children and older adults. While our current findings indicate negative behavioral correlates of shame in an emerging adult population, further study may indicate whether impulsivity interacts with self-conscious emotions across development. Lastly, the use of additional behavioral measures of risk-taking, such as the Balloon Analogue Risk Task (BART: Lejuez et al., 2002), may provide an alternative to self-report.

### 4.3 Conclusion

In conclusion, latent variable double mediation models indicate that impulsivity predicts both internalizing and externalizing behavior problems through its association with guilt. The self-report nature of behavior problems in this study makes it difficult to discern whether guilt-prone individuals truly show fewer problem behaviors or whether they are just less likely to report these behaviors. Guilt may make reporting maladaptive behaviors a more negative affective experience, so the relationship between these constructs should be further examined. Other possible mechanisms underlying health-risk behaviors in impulsive populations should be subjects of future analyses. Research into the possible protective qualities of shame and guilt may inform the development of behavioral interventions for impulsivity.

## Declarations

### Author contribution statement

H. Sanchez: Conceived and designed the experiments; Performed the experiments; Wrote the paper.

S. Fields: Conceived and designed the experiments; Analyzed and interpreted the data; Wrote the paper.

D. A. Clark: Analyzed and interpreted the data; Wrote the paper.

### Funding statement

This research did not receive any specific grant from funding agencies in the public, commercial, or not-for-profit sectors.

### Competing interest statement

The authors declare no conflict of interest.

### Additional information

No additional information is available for this paper.

## Footnotes

1 Models were also run in which impulsivity was included as a manifest composite variable (the average of the six standardized indicators). The conclusions drawn from this approach were analogous to those presented here.
